# Substance Abuse: Resurgence of Teen Inhalant Use

**DOI:** 10.1289/ehp.113-a808

**Published:** 2005-12

**Authors:** Ron Chepesiuk

The 2004 Monitoring the Future (MTF) survey showed that inhalant use (“huffing”) is rising among American teenage students, particularly 8th graders. The results, released in December 2004, showed that 9.6% of 8th graders used inhalants in 2004, up from 7.7% in 2002 and 8.7% in 2003. Inhalant use was also up slightly among 10th and 12th graders in 2004. Findings from the latest MTF will be released in late December 2005, and researchers are anxious to see if the trend holds.

“These increases are disturbing because they come after a long period of decline in inhalant use by students in all three grades,” says Lloyd D. Johnston, a professor at the University of Michigan Institute for Social Research and principal investigator of the MTF since it began in 1975. “We are concerned that the use of this class of drugs may be about to rebound.”

Each year, the MTF, which is funded under grants from the National Institute on Drug Abuse (NIDA), asks approximately 50,000 8th-, 10th-, and 12th-grade students in some 400 schools nationwide about their use of drugs, alcohol, and cigarettes. The data gathered are used to help government officials and policy makers identify potential drug problem areas so they can target resources to deal with them.

“We know that inhalant use starts early and that long-term abusers are among the most difficult drug abuse patients to treat,” says NIDA director Nora Volkow. “It is critical that research efforts to characterize the behavioral effects of inhalants intensify, so that more effective preventions, interventions, and treatments can be developed.” This year, NIDA announced the continuation of a broad-based research initiative begun in 2002 to address the epidemiologic, social, behavioral, cognitive, and neurobiological consequences of inhalant abuse, as well as treatment and prevention.

More than 1,000 readily available products are used as inhalants, and they can potentially kill, according to the Office of National Drug Control Policy (ONDCP). Such products include glue, shoe polish, gasoline, lighter fluid, and the propellants in spray deodorant, hair sprays, and canned whipped cream.

The ONDCP further reports that glue, shoe polish, and toluene-containing products were the most commonly abused inhalants among users aged 12 to 17. According to the American Association of Poison Control Centers, gasoline accounted for the greatest percentage (44%) of reported inhalant deaths between 1996 and 2001, followed by air fresheners (26%) and propane/butane (11%). Other health effects of inhalant use include headache, nausea, vomiting, slurred speech, loss of motor coordination, and wheezing.

The physical and social environment both play a key role in inhalant use, says Harvey Weiss, executive director of the National Inhalant Prevention Coalition. Treatment sometimes requires removing the abuser from the environment in which he or she is abusing inhalants. “We should not view inhalant abuse [simply] as a substance abuse problem,” Weiss says. “It’s a public health problem, so we need to do more public health outreach to young people.”

Sources believe that education is the key to preventing inhalant use from becoming a dangerous fad. When MTF data from the mid-1990s began showing a long-term gradual increase in inhalant use, the Partnership for a Drug-Free America and NIDA mounted an aggressive media campaign about the dangers of inhalants. The next round of MTF data showed a decline in inhalant use and a concurrent increase in young people viewing inhalants’ use as risky, but use began climbing again after the media campaign ended.

“Of course, the evidence is circumstantial, but we’ve seen the same thing happen for so many other drugs,” Johnston says. “A drug can have a resurgence in use among young people because of what I call ‘generation forgetting’—that is, a new generation of young people comes along that hasn’t heard too much about a drug, so it is naïve about the consequences of its use. That begins to change when a public education campaign is launched.”

Despite the MTF findings, the U.S. government hasn’t yet documented a trend indicating a rise in inhalant use among teenagers, says Terry Zobeck, deputy associate director for policy and budget at the ONDCP. But he adds, “The MTF is respected and well documented. We will be quite concerned if its next survey shows that inhalant use is up for the third year in a row.”

## Figures and Tables

**Figure:**
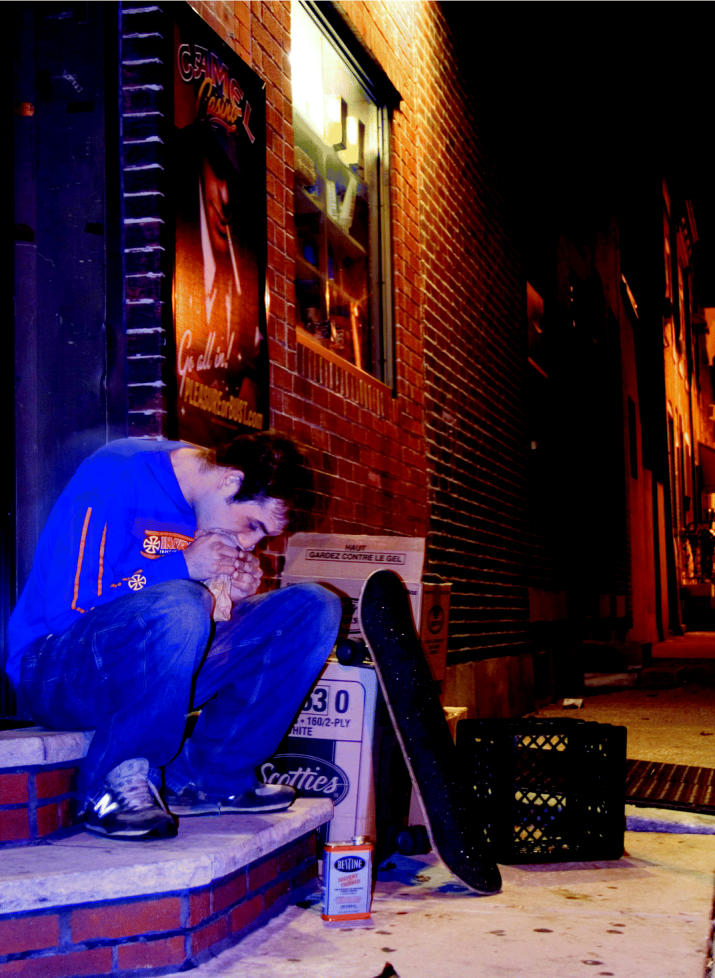
Huffing is up. A new survey shows the practice of inhaling toxic—often deadly—substances is increasing among American teens.

